# Exploring key factors in online shopping with a hybrid model

**DOI:** 10.1186/s40064-016-3746-4

**Published:** 2016-11-30

**Authors:** Hsiao-Ming Chen, Chia-Huei Wu, Sang-Bing Tsai, Jian Yu, Jiangtao Wang, Yuxiang Zheng

**Affiliations:** 1 School of Economics and Management, Shanghai Maritime University, Shanghai, 201306 China; 2 Department of Industrial Education, National Taiwan Normal University, Taipei, 106 Taiwan; 3 Institute of Service Industries and Management, Minghsin University of Science Technology, Hsinchu, 304 Taiwan; 4 Zhongshan Institute, University of Electronic Science and Technology of China, Zhongshan, Guangdong 528402 China; 5 Law School, Nankai University, Tianjin, 300071 China; 6 School of Business, Dalian University of Technology, Panjin, 124221 China; 7 Business School, Nankai University, Tianjin, 300071 China

**Keywords:** Analytic network process (ANP), DEMATEL-based ANP (DANP), E-business, Online shopping, Service quality, Management

## Abstract

**Introduction:**

Nowadays, the web increasingly influences retail sales. An in-depth analysis of consumer decision-making in the context of e-business has become an important issue for internet vendors. However, factors affecting e-business are complicated and intertwined.

**Case description:**

To stimulate online sales, understanding key influential factors and causal relationships among the factors is important. To gain more insights into this issue, this paper introduces a hybrid method, which combines the Decision Making Trial and Evaluation Laboratory (DEMATEL) with the analytic network process, called DANP method, to find out the driving factors that influence the online business mostly.

**Discussion and Evaluation:**

By DEMATEL approach the causal graph showed that “online service” dimension has the highest degree of direct impact on other dimensions; thus, the internet vendor is suggested to made strong efforts on service quality throughout the online shopping process.

**Conclusions:**

In addition, the study adopted DANP to measure the importance of key factors, among which “transaction security” proves to be the most important criterion. Hence, transaction security should be treated with top priority to boost the online businesses. From our study with DANP approach, the comprehensive information can be visually detected so that the decision makers can spotlight on the root causes to develop effectual actions.

## Introduction

As the significant share of the population goes online, the web increasingly influences retail sales. E-commerce revenues continue to climb up around the globe and the consumers are changing their consuming behaviors at the same time. According to Forrester Research, an independent technology and market research company, online retail sales in US reach $334 billion in 2015, approximately 10% of all sales in the US. E-commerce will experience a strong compound annual growth rate (CAGR) of 10% over the next 5 years, translating to $480 billion in online sales by 2019. In UK, the online sales are projected at a compound annual growth rate of 11%, from £30.1 billion in 2011 to £51.0 billion in 2016, and the proportion of online shoppers will increase from 75% of the population in 2011 to 85% in 2016. Similarly, the proportion of online shoppers in Sweden will increase from 72% of the population in 2011 to 86% in 2016 (Rigby 2012). Asia Pacific (China, Japan, South Korea, India, and Australia) contains both the largest and the fastest growing e-commerce markets, and total online retail revenues will nearly double from $733 billion in 2015 to $1.4 trillion in 2020; China, especially, is the biggest market and accounts for 80% of Asia Pacific online retail sales; it is expected to become the first market to reach $1 trillion in online retail sales in 2019, according to Forrester Research. As the competition among internet marketers increases, it is becoming increasingly important for online sellers to understand the factors that affect the attitudes of individual customers toward online purchase.

Past researches focused on understanding the consumer’s purchase behaviors via the internet (Akhter 2012; Chiou and Ting 2011; Chiu et al. 2013; Kim et al. 2012a; Overby and Lee 2006; Teo and Yu 2005). Several studies identified the key factors leading to a success of e-business (Kim et al. 2012b); many others explored the influences on online shoppers’ evaluations regarding repurchase from an e-store (Chiu et al. 2009; Gupta and Kim 2007; Kim et al. 2012b), customer satisfaction (Dong 2012) and intentions linked to complaints (Hong and Kim 2012; Wu 2012). Another part of researchers consider e-service quality and online services in general are recognized to be a crucial determinant in building competitive advantage (Chiou et al. 2011; Cebi 2013; Tontini 2016; Kim and Lee 2004; Lin 2011; Santos 2003) and relates to measures customer intention to buy (Bai et al. 2008). In addition, the technique of multiple regression was commonly used to show the effects of interaction between online shopping motivation and product type (Chiou and Ting 2011); structural equation modeling (SEM) approach was also frequently used to test the hypothetical relations between independent and dependent variables (Hong and Kim 2012; Kim et al. 2012b; Wu 2012); the importance performance analysis (IPA) attempted to identify what should be improved or offered on websites and in online services (Dong 2012; Oh and Zhang 2010), and many studies on the acceptance of online shopping have been done under the logic of the attitudinal model, particularly TRA and its two extensions TPB and TAM (Ajzen 1991; Davis 1989; Fishbein and Ajzen 1975). These studies have made important contribution to the understanding of dynamics of e-business. However, consumers need consideration of multiple criteria, but few of previous researches discussed on the intertwined interactions and causal relationships, even in lack of the interdependence and feedback among the criteria during the evaluation process, and do not focus on different weights of criteria; even the online consumers’ perception study (Kim et al. 2011) from top journals, the weight of criteria in MDS has remained equal, but the equal-weighting in calculating is not that reasonable in the real world situations. Therefore, this paper proposes an approach that combines the Decision Making Trial and Evaluation Laboratory (DEMATEL) method with the analytic network process (ANP) method, called DANP, to address the problems of interdependence and feedback and weighting measure (Liu et al. 2012). Thus, the DANP method can provide valuable information and some worthwhile recommendations for achieving a competitive advantage.

 The rest of this paper is organized as follows. The second section is the literature review. In third section, the DANP method is described. In fourth section, an empirical study is to demonstrate usefulness of the proposed method. Finally, conclusions and suggestions for future studies are addressed.

## Literature review

For ordinary buyers, the internet serves as a convenient shopping medium that can offer such benefits as saving time and effort, less transportation and search costs, no waiting lines, improved shopping enjoyment, precise price comparison, collection of data, and location of information, convenient information acquiring, and subsequent ability to search more frequently and intensely, and the chance for buyers to design products and services according to their own needs and preferences (Chiou and Ting 2011; Fred and Thatcher 2010; Forsythe and Shi 2003; Ha and Stoel 2009; Kim et al. 2012a, 2009; Lee et al. 2011; Liao et al. 2009; Rozenn and Thierry 2013; Zwass 1991). In general, it gives consumers the ability to shop from their home for a variety of products or services anytime from all over the world. Most of these factors have positive effects towards online shopping intention and behavior.

In practice, however, many consumers still hesitate to shop online mainly due to some risks, as well as information privacy, security issues and credit-card concerns. Risks may arise because online shoppers do not have the opportunity to interact with sellers directly and to examine the products before making a payment. They would perceive a higher risk for shopping online along with their purchase intention that has consequently been influenced. Thus, a number of studies have pointed out that the negative factors in the purchase process can be basically characterized as twofold: perceived risk and uncertainty (Crespo and del Bosque 2010; Forsythe and Shi 2003; Ha and Stoel 2009; Kim et al. 2012a). Past researches observed that perceived risk plays an important role in online shopping behavior. Perceived risk occurs in an online transaction when the consumer is required to provide personal and credit card information before they buy anything (Akhter 2012). Concerns over privacy and credit card security problems may emerge in the course of internet transactions. Concerns about privacy are, in fact, a major factor that negatively affects purchasing behavior, and it can play a critical role in consumer decision-making. In addition, uncertainty has been shown to exert a heavy influence on the purchase decision (Forsythe and Shi 2003). Having concerns over possible unforeseen purchasing results, the consumer worries that products may fail to satisfy consumers’ expectation, fail to provide the desired benefits, or may even not function properly (Chang and Tseng 2013; Hong and Kim 2012). We suggest that perceived risk of online purchase should be explicitly examined in research of online shopping behavior.

Moreover, trust often plays a key role in consumer adoption of online shopping (Kim et al. 2010; Cho 2010) and lack of trust in e-retails has been identified as one of the greatest barriers inhibiting internet transactions—a major reason that many people have not yet made the decision to shop online (Ha and Stoel 2009; Kim et al. 2012a). Indeed, trust is an essential factor in the transactional relationship, and e-environmental uncertainties between internet vendors and consumers are of primary concern (Chang et al. 2005; Chiu et al. 2006; Seyed-Hosseini et al. 2006; Shahraki and Paghaleh 2011).

## Methods

**Fig. 1:**
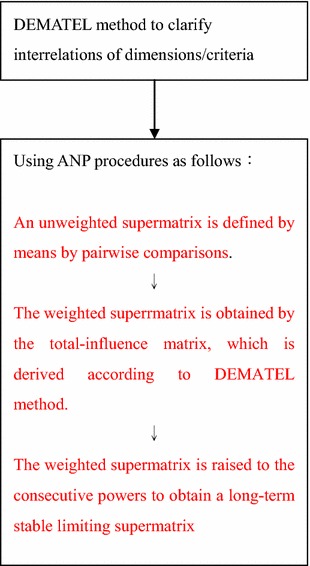
DANP procedure (Ou Yang et al. 2008)

This paper introduces a hybrid method that combines the DEMATEL with the ANP to confirm the effects of intertwined criteria and to measure their importance. The DEMATEL method and the ANP method are briefly introduced, and the detailed DANP procedures are schematically shown in Fig. 1 and elaborated as follows (Fig. 1).

### DEMATEL

Developed by the Geneva Research Centre of the Battelle Memorial Institute, the DEMATEL technique is a mathematical procedure to obtain the direct and indirect causation as well as the influential strength across quality features by applying the matrix computation to complex systems and comparing the interrelations across quality features (Gabus and Fontela 1973; Fontela and Gabus 1976; Tsai 2016; Tsai et al. 2016b; Zhou et al. 2016). The DEMATEL technique converts complex systems to a clear causal structure that simplifies the interrelationship across quality features of complex systems into cause group and effect group; therefore, it helps locate the causal factors and improvement of complex systems via the degree of interrelations across quantified quality features (Tzeng et al. 2007; Wu and Lee 2007; Sun 2013; Ting et al. 2013; Wang and Dong 2015). In particular, the visually structural matrix and causal figures expressing the causation and affecting levels across quality features of complex systems have been proven of great use for decision making (Tsai and Xue 2013; Lee et al. 2014a, b; Guo and Tsai 2015; Guo et al. 2015).

In recent years, DEMATEL has been widely applied to various fields. For example, in causal analytic method for group decision making (Lin and Wu 2008; Tsai et al. 2014; Qu et al. 2015; Tsai et al. 2015, 2016a), safety management system for airlines (Liou et al. 2007), selecting management systems of SMEs (Tsai and Chou 2009), evaluating users’ behavioral intention to use a new etching plasma technology (Lee et al. 2010), evaluating performance criteria of employment service outreach program personnel (Wu et al. 2010), and exploring the core competences and causal effect of the IC design service company (Lin et al. 2011b), etc. In addition, it has been integrated with the other methods, such as analytic network process (ANP) while selecting knowledge management strategies (Wu 2008); a back-propagation artificial neural network while conducting importance-performance analysis (Hu et al. 2009); ANP, and zero–one goal programming (Tsai and Chou 2009); and ANP to form an integrated MCDM technique to weight attribute clusters (Yang and Tzeng 2011). The method can be summarized as follows.

- *Step 1* Generating the direct-relation matrix *X*

Measuring the relationship between criteria requires that the comparison scale is designed with a number of levels. We have decided to use five to use in this case: from 0 (no influence), 1 (very low influence), 2 (low influence), 3 (high influence) to 4 (very high influence). Assuming that there are n criteria that influence a complex system, the n criteria can be extended as an n × n direct-relation matrix (*X*) based on mutual influence relationships and levels of influence and using the expert opinion method. In the direct relationship matrix *X*, *X*_*ij*_ is denoted as to the degree which the criterion *i* affects the criterion *j*.

1 $$X = \left[ {\begin{array}{*{20}l} 0 \hfill & {x_{12} } \hfill & \cdots \hfill & {x_{1n} } \hfill \\ {x_{21} } \hfill & 0 \hfill & \cdots \hfill & {x_{2n} } \hfill \\ \vdots \hfill & \vdots \hfill & \ddots \hfill & \vdots \hfill \\ {x_{n1} } \hfill & {x_{n2} } \hfill & \cdots \hfill & 0 \hfill \\ \end{array} } \right]$$

- *Step 2* Normalizing the direct-relation matrix *N*

Then, the normalization of direct-relation matrix *X* should be taken into account. Regarding the calculation of the normalized direct-relation matrix (*N*), Kim (2006), Lin and Wu (2008) and Lee et al. (2010) utilized the maximum sum of the row vector as the normalization baseline.

2 $${\text{Definition:}}\quad \lambda = \frac{1}{{\begin{array}{*{20}c} {Max} \\ {1 \le i \le n} \\ \end{array} \left( {\sum\nolimits_{j = 1}^{n} {x_{ij} } } \right)}}$$

Subsequently, *X* was multiplied by λ, and *N* was acquired.

3 $$N = \lambda X$$

- *Step 3* Attaining the total-relation matrix *T*

Once the normalized direct-relation matrix *N* is obtained, the total relation matrix *T* can be acquired by the means of Eq. (4), in which the *I* is denoted as the identity matrix.

4 $$T = \mathop {\lim }\limits_{k \to \infty } \left( {N + N^{2} + \cdots + N^{k} } \right) = N\left( {I - N} \right)^{ - 1}$$

The sum of rows and the sum of columns are contained in vector D and vector R, respectively. Components of both vectors are obtained by means of Eqs. (5) and (6). Then, the horizontal axis vector (*D* + *R*) named “Prominence” is made by adding *R* to *D*, which reveals how much importance the criterion has. Similarly, the vertical axis (*D* − *R*) named “Relation” is made by subtracting *R* from *D*, which may divide criteria into a cause group and an effect group. Generally, when (*D* − *R*) is positive, the criterion belongs to the cause group; when the (*D* − *R*) is negative, the criterion belongs to the effect group. Therefore, the causal diagram can be acquired by mapping the dataset of both indices, which will provide valuable insight for making decisions.

5 $$D_{i} = \sum\limits_{j = 1}^{n} {t_{ij} } \quad \left( {i = 1,2, \ldots ,n} \right)$$

6 $$R_{j} = \sum\limits_{i = 1}^{n} {t_{ij} } \quad \left( {j = 1,2, \ldots ,n} \right)$$

- *Step 4* Building a causal map

To express a complex problem by a simplified visual map, locate the figures of coordinates (*D* + *R, D* − *R*) by employing the prominence (*D* + *R*) as a horizontal axis and the relation (*D* − *R*) as a vertical axis. As such, a two-dimensional causal map can be built in four quadrants. The items located in quadrant I (large prominence, positive relation) represent the causal urgent items, which require improvement in a “direct” manner with top priority as they serve as the driving factors. The items located in quadrant IV (large prominence, negative relation) represent the effect urgent items, which also require improvement but in an “indirect” manner with high priority as they are affected by others. In contrast, the items located in quadrant II (small prominence, positive relation) are not the major items, yet one may carry out a “direct” improvement if the resources are sufficiently available. Finally, the items located in quadrant III (small prominence, negative relation) are not the major items. They are affected by others, thus calling for an “indirect” improvement with the lowest priority.

### ANP

The analytic hierarchy process (AHP) has been widely used for analyzing complex decision problems since it was developed by Thomas L. Saaty in the 1970s. Each element in the AHP hierarchy is assumed to be independent of one another. In many real world decision situations, however, the elements are most likely intertwined and interdependent; thus the AHP is not appropriate (Lan et al. 2013). To transcend the limits of the AHP, the ANP is developed. It utilizes the super-matrix approach. The first difference is that the AHP is a special case of the ANP, because the ANP handles dependence within a cluster (inner dependence) and among different clusters (outer dependence). Secondly, the ANP utilizes a nonlinear structure, while the AHP applies a hierarchical and linear structure with a goal at the top level and the alternatives in the bottom level (Saaty 1996). The ANP provides a way to input judgments and measurements to derive ratio scale priorities for the distribution of influence among the criteria and groups of criteria in the decision making process (Chen et al. 2011). The method has been applied successfully while solving many practical decision-making problems, such as project selection, product planning, green supply chain management and optimal scheduling problem (Meade and Presley 2002; Lee and Kim 2000; Karsak et al. 2002; Sarkis 2003; Momoh and Zhu 2003). Thus the ANP method has partially resolved the issues of dependence among dimensions and the self-feedback effect within a dimension. However, there are two limitations remaining: (1) the plausible interrelationship between any two criteria that belong to different dimensions and (2) the weighted supermatrix is calculated by assuming the equal weight in all dimensions so that each column sums to unity (Hsu et al. 2012).

### Procedures for the DANP method

When dealing with ANP, our use of the normalization method implies that each cluster has the same weight. However, there are different degrees of influence among the clusters of factors/criteria in the world. Thus, the assumption of equal weights of each cluster to obtain the weighted super-matrix is unrealistic and needs to be improved. Therefore, this paper combines the DEMATEL with the ANP, called DEMATEL-based ANP method, or DANP proposed to avoid the shortcomings mentioned in the ANP. The DANP method inherits the advantage from DEMATEL by allowing the interrelationships among all criteria; in addition, the dimensional weights obtained by DEMATEL can relax the equal-weight assumption in ANP; the weighted super-matrix can thus be adjusted (by DEMATEL) to have the final DANP influential weights for all criteria (Ou Yang et al. 2013). We expect not only to deal with the problem of interdependence and feedback but also improve the normalized super-matrix to derive the relative influential weights in dimensions/criteria. The DANP can reflect real world situations more accurately (Ou Yang et al. 2008) and can provide valuable information for decision making. It has been successfully applied to solve a variety of MCDM problems, such as improving marketing (Chiu et al. 2013), tourism policy (Liu et al. 2012), airline partner selection (Liou et al. 2011), information security risk (Ou Yang et al. 2013; Lo and Chen 2012), environment watershed plans (Chen et al. 2010).

DANP is therefore more suitable in the real world. The procedure of our proposed method is mainly divided into four steps (Fig. 1) and can be explained briefly by Hsu et al. (2012) and Ou Yang et al. (2008).

*Step 1* The total relation matrix *T* will be obtained from DEMATEL. Each column of the matrix sums up to unity. The matarix is shown in Eq. (7):7


*Step 2* Next, normalize *T*_*c*_ with the total degree of influence and obtain $T_{c}^{\alpha }$, as shown in Eq. (8).8
Then, normalize $T_{c}^{\alpha 11}$ via Eq. (9), and repeat to obtain $T_{c}^{\alpha nn}$. Let us take matrix $T_{c}^{\alpha 11}$ as an example. It is normalized in a way which is presented in Eq. (9); all other matrices follow a similar normalization scheme.

9 $${\mathbf{T}}_{c}^{\alpha 11} = \left[ {\begin{array}{ccccc} {t_{c}^{11} /d_{1}^{11} } \hfill & \cdots \hfill & {t_{c1j}^{11} /d_{1}^{11} } \hfill & \ldots \hfill & {t_{{c^{{1m_{1} }} }}^{11} /d_{1}^{11} } \hfill \\ \vdots \hfill & {} \hfill & \vdots \hfill & {} \hfill & \vdots \hfill \\ {t_{{c^{i1} }}^{11} /d_{i}^{11} } \hfill & \ldots \hfill & {t_{{c^{ij} }}^{11} /d_{i}^{11} } \hfill & \cdots \hfill & {t_{{c^{{im_{1} }} }}^{11} /d_{i}^{11} } \hfill \\ \vdots \hfill & {} \hfill & \vdots \hfill & {} \hfill & \vdots \hfill \\ {t_{{c^{{m_{1}^{1} }} }}^{11} /d_{{m_{1} }}^{11} } \hfill & \cdots \hfill & {t_{{c^{{m_{1}^{j} }} }}^{11} /d_{{m_{1} }}^{11} } \hfill & \cdots \hfill & {t_{{c^{{m_{1} m_{1} }} }}^{11} /d_{n} } \hfill \\ \end{array} } \right] = \left[ {\begin{array}{ccccc} {t_{{c^{11} }}^{\alpha 11} } & \cdots & {t_{{c^{1j} }}^{\alpha 11} } & \cdots & {t_{{c^{{1m_{1} }} }}^{\alpha 11} } \\ \vdots & {} & \vdots & {} & \vdots \\ {t_{{c^{i1} }}^{\alpha 11} } & \cdots & {t_{{c^{ij} }}^{\alpha 11} } & \cdots & {t_{{c^{{im_{1} }} }}^{\alpha 11} } \\ \vdots & {} & \vdots & {} & \vdots \\ {t_{{c^{{m_{1}^{1} }} }}^{\alpha 11} } & \cdots & {t_{{c^{{m_{1}^{j} }} }}^{\alpha 11} } & \cdots & {t_{{c^{{m_{1} m_{1} }} }}^{\alpha 11} } \\ \end{array} } \right]$$

The total effect matrix is normalized into the super-matrix according to the dependence relationships in the group. This allows us to obtain the unweighted super-matrix, as shown in Eq. (10).10


*Step 3* Obtain the weighted super-matrix by deriving the matrix of the total effect of dimensions $T_{D}^{{}}$. Then, $T_{D}^{{}}$ can be normalized to become $T_{D}^{\alpha }$ by Eq. (11):

11 $${\mathbf{T}}_{D}^{\alpha } = \left[ {\begin{array}{*{20}c} {t_{D}^{11} /d_{1} } & \cdots & {t_{D}^{1j} /d_{1} } & \cdots & {t_{D}^{1n} /d_{1} } \\ \vdots & {} & \vdots & {} & \vdots \\ {t_{D}^{i1} /d_{2} } & \cdots & {t_{D}^{ij} /d_{2} } & \cdots & {t_{D}^{in} /d_{2} } \\ \vdots & {} & \vdots & {} & \vdots \\ {t_{D}^{n1} /d_{n} } & \cdots & {t_{D}^{nj} /d_{n} } & \cdots & {t_{D}^{nn} /d_{n} } \\ \end{array} } \right] = \left[ {\begin{array}{*{20}c} {t_{D}^{\alpha 11} } & \cdots & {t_{D}^{\alpha 1j} } & \cdots & {t_{D}^{\alpha 1n} } \\ \vdots & {} & \vdots & {} & \vdots \\ {t_{D}^{\alpha i1} } & \cdots & {t_{D}^{\alpha ij} } & \cdots & {t_{D}^{\alpha in} } \\ \vdots & {} & \vdots & {} & \vdots \\ {t_{D}^{\alpha n1} } & \cdots & {t_{D}^{\alpha nj} } & \cdots & {t_{D}^{\alpha nn} } \\ \end{array} } \right]$$

Then, the normalized $T_{D}^{\alpha }$ is transformed into the unweighted super-matrix $W$ to obtain the weighted super-matrix $W_{c}^{*}$, as shown in Eq. (12):

12 $$W^{\alpha } = \varvec{T}_{D}^{\alpha } W = \left[ {\begin{array}{*{20}c} {t_{D}^{\alpha 11} \times \varvec{W}^{11} } & {t_{D}^{\alpha 21} \times \varvec{W}^{21} } & \cdots & \cdots & {t_{D}^{\alpha n1} \times \varvec{W}^{n1} } \\ {t_{D}^{\alpha 12} \times \varvec{W}^{12} } & {t_{D}^{\alpha 22} \times \varvec{W}^{22} } & \vdots & {} & \vdots \\ \vdots & \cdots & {t_{D}^{\alpha ij} \times \varvec{W}^{ij} } & \cdots & {t_{D}^{\alpha nj} \times \varvec{W}^{nj} } \\ \vdots & {} & \vdots & {} & \vdots \\ {t_{D}^{\alpha 1n} \times \varvec{W}^{1n} } & {t_{D}^{\alpha 2n} \times \varvec{W}^{2n} } & \cdots & \cdots & {t_{D}^{\alpha nn} \times \varvec{W}^{nn} } \\ \end{array} } \right]$$

*Step 4* Obtain the limiting supermatrix. According to the weighted supermatrix $W^{\alpha }$, it multiplies by itself multiple times to obtain a limiting supermatrix. Then, the influential weights of each criterion can be obtained from $\mathop {\lim }\limits_{z \to \infty } (W^{\alpha } )^{z}$, where $z$ represents any number for power.

## Example

An empirical example for exposing the driving factors affecting online business is illustrated to demonstrate the purpose method to be more rational and suitable in this section, which is divided into three subsections: (1) identification of criteria and dimensions, (2) calculating the weights of criteria, (3) discussion.

### Identification of criteria and dimensions

First, we designed a questionnaire to gather information from experts with professional knowledge and experience. Furthermore, the background of experts is described as follows: three scholars of marketing specialize in the management of marketing and teaching marketing course in a university; ten made regular purchases from internet shop at least 30 times per year and whose names had been registered in the customer database; seven had been involved in managing e-shopping stores operations on the Yahoo website over 5 years. This site has been the most popular website for more than 3 years (Chiu et al. 2013); it offers a wide range of products, including bags, clothing, fashion, cosmetics, sporting goods and many other items. The demographic distribution was as follows: 13 females and 7 males; 100% bachelor’s degree; age around 40 years old (30% less than 40, and 70% 40 or more). Each interview with an expert took approximately 30–40 min to finish the questionnaire.

By referring to the factors affecting e-business from relevant literatures as aforementioned, then after several group meetings, those who were interviewed decided to refer to some researchers (e.g., Akhter 2012; Cebi 2013; Kim et al. 2012a) by selecting the perceived benefits (acting as motivators): online service (responsiveness, communication and interaction and reliability) and conveniences (provides better prices, time saving and wider selections). Moreover, they also chose perceived risks (acting as barriers) from a recent work (e.g., Akhter 2012; Cebi 2013; Dickinger and Stangl 2013; Hong and Yi 2012), which included trust and risk (trust, privacy risk and transactions security), and uncertainty (performance risk, product risk and quality of product) over purchasing outcomes. The 12 criteria and their meaning are depicted in Table 1.

**Table 1:** The influence dimensions and criteria on e-businesses used in the case study

Dimensions		Criteria		Meanings for the criteria	References
A	Online service	a1	Responsiveness	Speed and accuracy of response	Cebi (2013), Dickinger and Stangl (2013)
		a2	Communication and interaction	Possibility of communication between customers	Cebi (2013), Dickinger and Stangl (2013)
		a3	Reliability	Supplying as promised; correct technical functioning of the site	Cebi (2013), Dong (2012)
B	Convenience	b1	Provides better prices	Obtaining more information about price and comparing for the best price through the Website	Kim et al. (2012a), Chang and Tseng (2013), Lin et al. (2011a), Close and Kinney (2010)
		b2	Time saving	Time and effort savings; products available all the time	Kim et al. (2012b), Hernández et al. (2010)
		b3	Wider selections	Offering more useful information about the choices available through internet	Wong et al. (2012), Chiou and Ting (2011)
C	Trust and Risk	c1	Trust	A set of beliefs about the trustworthiness of an internet vendor, like dependability of online stores and privacy policy	Cebi (2013), Choi et al. (2013), Kim et al. (2012a)
		c2	Privacy risk	A concern when providing and sending personal or financial information	Lian and Lin (2008), Akhter (2012)
		c3	Transaction security	Providing credit card information; safety of use of credit cards	Hong and Yi (2012), Lee (2009)
D	Uncertainty	d1	Performance risk	Product may not perform as expected	Hong and Yi (2012), Wu (2012)
		d2	Product risk	Risk of non-delivery of goods after payment	Hong and Yi (2012), Lin et al. (2011a)
		d3	Quality of product	Lack of any guarantee of quality of goods and sold	Wu (2012); Dickinger and Stangl (2013)

### Calculating the weights of criteria

For the measurement of relationship, the experts were asked to determine the influential importance among the 12 criteria with a scale ranging suggested by Wu et al. (2011) and consisting of five respective levels from 0 (no influence), 1 (very low influence), 2 (low influence), 3 (high influence) to 4 (very high influence). In addition, the measurement scale is also divided into 0, 1, 2, 3, 4, and 5 level, which respectively represent “no impact”, “very low impact” “low impact”, “medium impact” “high impact” and “great impact” (Kim 2006), and Huang et al. (2007), adopted 11 levels, 0, 1,…, 10, from “no impact” to “great impact”. As viewed, the decision of measurement scale imposes not any special constraint or regulation (Hu et al. 2009).

Then the average initial direct-relation matrix *X* can be obtained by pairwise comparison in terms of influences direction and intensity. The questionnaire results are presented in Table 2.

**Table 2:** The initial direct-relation matrix X

Criteria	a1	a2	a3	b1	b2	b3	c1	c2	c3	d1	d2	d3
a1	4.0	3.2	0.4	3.0	1.2	1.8	0.2	0.6	1.0	1.6	1.4	4.2
a2	4.0	3.4	0.4	1.8	1.2	3.0	1.0	0.2	2.0	1.8	1.8	3.5
a3	3.0	3.6	0.2	2.2	1.4	3.2	3.8	3.8	2.4	2.4	1.6	1.2
b1	0.4	0.6	1.0	1.8	2.6	0.4	0.2	0.8	1.6	1.0	1.2	0.0
b2	3.0	1.0	1.0	0.8	1.6	0.4	0.4	0.4	1.2	0.8	0.8	0.0
b3	0.8	1.0	1.2	3.2	2.6	0.4	0.0	0.0	0.6	0.6	0.8	3.2
c1	1.8	2.8	2.8	0.4	1.4	0.8	2.6	2.4	2.8	2.4	2.6	1.3
c2	1.0	1.2	3.2	0.0	0.0	0.0	3.6	3.0	0.4	0.2	0.2	1.4
c3	0.4	0.0	2.6	0.0	0.8	0.0	3.6	3.6	1.0	0.2	0.2	0.0
d1	1.0	0.8	1.8	0.6	0.2	0.8	2.4	1.2	1.0	1.6	2.4	0.0
d2	0.8	1.0	2.4	0.6	0.0	0.0	2.8	1.2	1.8	2.4	1.8	3.5
d3	1.2	1.8	3.2	1.2	0.4	0.6	3.8	0.6	0.6	2.4	1.8	0.0

The normalized direct-relation matrix can be obtained through the formulae (2) and (3); then the total-relation matrix can be acquired by the formula (4). The total-relation matrix is presented in Table 3.

**Table 3:** The total-relation criteria matrix $Tc$

Criteria	a1	a2	a3	b1	b2	b3	c1	c2	c3	d1	d2	d3
a1	0.28	0.25	0.13	0.21	0.13	0.14	0.15	0.11	0.13	0.17	0.16	0.27
a2	0.29	0.27	0.14	0.18	0.13	0.19	0.19	0.11	0.18	0.19	0.18	0.26
a3	0.28	0.30	0.17	0.20	0.15	0.21	0.32	0.27	0.22	0.22	0.19	0.20
b1	0.08	0.08	0.09	0.10	0.13	0.05	0.08	0.08	0.11	0.08	0.09	0.05
b2	0.18	0.11	0.09	0.08	0.10	0.06	0.08	0.07	0.09	0.08	0.08	0.07
b3	0.11	0.11	0.11	0.17	0.14	0.06	0.09	0.06	0.08	0.09	0.09	0.17
c1	0.22	0.25	0.24	0.12	0.13	0.12	0.28	0.22	0.22	0.21	0.21	0.18
c2	0.14	0.15	0.21	0.07	0.05	0.06	0.25	0.20	0.10	0.09	0.09	0.14
c3	0.10	0.09	0.17	0.05	0.07	0.05	0.24	0.22	0.10	0.07	0.07	0.07
d1	0.12	0.12	0.15	0.08	0.06	0.08	0.19	0.12	0.11	0.13	0.16	0.08
d2	0.14	0.16	0.20	0.10	0.06	0.07	0.25	0.15	0.16	0.19	0.16	0.22
d3	0.16	0.19	0.22	0.12	0.08	0.09	0.27	0.13	0.12	0.19	0.16	0.10

The (*D* + *R*) and (*D* − *R*) values can be calculated from the total relation matrix (Table 3), and the (*D* + *R*) value indicates how important a criterion is, and it provides an index of the strength of influences given and received. The (*D* − *R*) value, on the other hand, indicates the size of the direct impact of this criterion on other criteria.

According to Table 4, when the relation of a criterion is *D* − *R* > 0, it means that it has a higher impact. Higher impact represents higher importance and should thus be considered first. If the relation of a criterion is *D* − *R* < 0, it means that it is influenced by other criteria. Here, the positive and negative key determinants are respectively displayed. We can thus clearly see that the cause (influencing) criteria consist of a1 (Responsiveness), a2 (Communication and interaction), a3 (Reliability), b3 (Wider selections). It is thus clear that strong efforts should be made to eliminate the influence of these criteria throughout the online shopping process. Take the impact of above-mentioned causal relationship on the influence of improvement decision making, the importance of these criteria can be prioritized as c1(Trust) > c3(Transaction security) > a1(Responsiveness) > c2(Privacy risk) > a2(Communication and interaction) > a3(Reliability) > b3(Wider selections) based on (*D* + *R*) values. The value of c1(Trust) is the greatest of all values, indicating that is viewed by the experts as the foremost driving factor, and thus is identified as the target for prioritized treatment in order to boost the online business.

**Table 4:** The total influence given and received by criteria

Criteria		*D*	*R*	*D* + *R*	*D* − *R*
a1	Responsiveness	2.244	0.880	3.124	1.363
a2	Communication and interaction	2.123	0.950	3.074	1.173
a3	Reliability	1.846	1.036	2.883	0.810
b1	Provides better prices	1.076	0.774	1.850	0.302
b2	Time saving	1.168	0.802	1.970	0.366
b3	Wider selections	1.679	0.996	2.676	0.683
c1	Trust	1.132	2.618	3.750	−1.486
c2	Privacy risk	0.999	2.131	3.131	−1.132
c3	Transaction security	0.848	2.700	3.548	−1.851
d1	Performance risk	0.626	0.471	1.097	0.155
d2	Product risk	0.941	1.148	2.089	−0.206
d3	Quality of product	1.041	1.217	2.258	−0.176

**Fig. 2:**
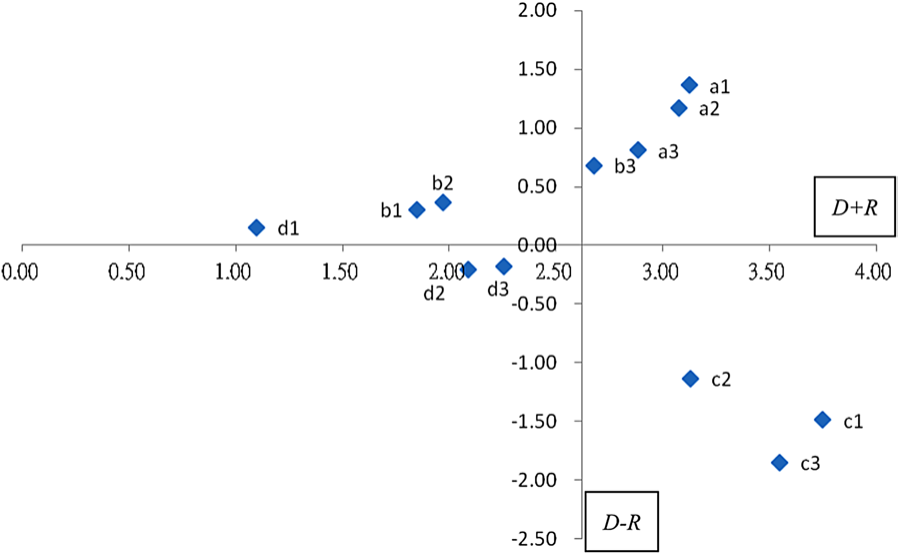
Causal map of relation within criteria

The cause group comprises the influencing criteria, whereas the effect group contains the influenced factors that are recipients of various influences. From the results, it appears that a successful e-retailer requires a high level of focus on the cause group (a1, a2, a3, b3, b2, b1, d1) rather than the effect group (c1, c3, c2, d3, d2) (Fig. 2).

Furthermore, the unweighted super-matrix is presented in Table 5, and the weighted super-matrix is presented in Table 6. Finally, the limiting supermartix is derived. Obtained final weights for the criteria are presented in Table 7.

**Table 5:** The unweighted super-matrix $Wc$

Unweighted	a1	a2	a3	b1	b2	b3	c1	c2	c3	d1	d2	d3
a1	0.032	0.066	0.061	0.043	0.051	0.051	0.069	0.060	0.071	0.058	0.058	0.072
a2	0.073	0.037	0.066	0.035	0.040	0.055	0.063	0.081	0.064	0.039	0.084	0.086
a3	0.064	0.067	0.043	0.099	0.086	0.071	0.062	0.053	0.060	0.108	0.064	0.048
b1	0.047	0.058	0.062	0.028	0.027	0.037	0.057	0.050	0.049	0.091	0.034	0.035
b2	0.062	0.051	0.073	0.067	0.028	0.053	0.054	0.046	0.041	0.020	0.034	0.036
b3	0.078	0.078	0.052	0.035	0.076	0.041	0.061	0.077	0.083	0.035	0.078	0.075
c1	0.161	0.161	0.156	0.189	0.192	0.197	0.104	0.160	0.160	0.183	0.160	0.177
c2	0.127	0.117	0.118	0.143	0.158	0.159	0.153	0.081	0.196	0.107	0.141	0.152
c3	0.146	0.157	0.160	0.207	0.189	0.183	0.193	0.210	0.094	0.207	0.195	0.167
d1	0.026	0.025	0.035	0.044	0.032	0.022	0.042	0.040	0.055	0.034	0.013	0.023
d2	0.086	0.094	0.105	0.057	0.061	0.062	0.060	0.064	0.080	0.061	0.030	0.071
d3	0.096	0.089	0.068	0.052	0.060	0.069	0.081	0.079	0.048	0.056	0.108	0.057

**Table 6:** The weighted super-matrix $W^{\alpha }$

Weighted	a1	a2	a3	b1	b2	b3	c1	c2	c3	d1	d2	d3
a1	0.0609	0.0609	0.0609	0.0609	0.0609	0.0609	0.0609	0.0609	0.0609	0.0609	0.0609	0.0609
a2	0.0642	0.0642	0.0642	0.0642	0.0642	0.0642	0.0642	0.0642	0.0642	0.0642	0.0642	0.0642
a3	0.0639	0.0639	0.0639	0.0639	0.0639	0.0639	0.0639	0.0639	0.0639	0.0639	0.0639	0.0639
b1	0.0483	0.0483	0.0483	0.0483	0.0483	0.0483	0.0483	0.0483	0.0483	0.0483	0.0483	0.0483
b2	0.0477	0.0477	0.0477	0.0477	0.0477	0.0477	0.0477	0.0477	0.0477	0.0477	0.0477	0.0477
b3	0.0678	0.0678	0.0678	0.0678	0.0678	0.0678	0.0678	0.0678	0.0678	0.0678	0.0678	0.0678
c1	0.1587	0.1587	0.1587	0.1587	0.1587	0.1587	0.1587	0.1587	0.1587	0.1587	0.1587	0.1587
c2	0.1414	0.1414	0.1414	0.1414	0.1414	0.1414	0.1414	0.1414	0.1414	0.1414	0.1414	0.1414
c3	0.1699	0.1699	0.1699	0.1699	0.1699	0.1699	0.1699	0.1699	0.1699	0.1699	0.1699	0.1699
d1	0.0361	0.0361	0.0361	0.0361	0.0361	0.0361	0.0361	0.0361	0.0361	0.0361	0.0361	0.0361
d2	0.0692	0.0692	0.0692	0.0692	0.0692	0.0692	0.0692	0.0692	0.0692	0.0692	0.0692	0.0692
d3	0.0719	0.0719	0.0719	0.0719	0.0719	0.0719	0.0719	0.0719	0.0719	0.0719	0.0719	0.0719

**Table 7:** The limiting DANP supermatrix

Criteria		DANP weights	Rank
a1	Responsiveness	0.0609	9
a2	Communication and interaction	0.0642	7
a3	Reliability	0.0639	8
b1	Provides better prices	0.0483	10
b2	Time saving	0.0477	11
b3	Wider selections	0.0678	6
c1	Trust	0.1587	2
c2	Privacy risk	0.1414	3
c3	Transaction security	0.1699	1
d1	Performance risk	0.0361	12
d2	Product risk	0.0692	5
d3	Quality of product	0.0719	4

As seen in the Table 7, results showed that experts were most concerned with transaction security (0.1699), trust (0.1587) and privacy risk (0.1414), which should be given priority to be improved in this empirical case. The “Trust and security” dimension plays an important role in e-business-one. It may heavily influence online consumers’ purchasing decisions. In contrast, the lowest priority is performance risk (0.0361). This finding indicates that managers may pay less attention to this factor, compared to the other criteria analyzed, when making efforts to promote e-business.

## Discussion

With an ever-increasing popularity of online shopping nowadays, an in-depth analysis of consumer decision-making in the context of e-business has become an important issue for internet vendors (Lee and Wu 2014). This study is intended to provide an in-depth understanding of the factors involved in satisfying customers’ needs, and thus to help managers initiate more effective marketing strategies to promote the growth of e-business.

Based on the results, some implications are discussed. Referring to Fig. 1, the horizontal axis (*D* + *R*), named “Prominence”, reveals how much importance the factor has; the vertical axis (*D* − *R*), named “Relation”, divides factors into a cause group and an effect group. The factor belonging to “cause group” if (*D* − *R*) is positive, whereas it belongs to “effect group” if (*D* − *R*) is negative. Therefore, we can determine the “Responsiveness” should be first to get improved. Second is “Communication and Interaction”. This is because they both influence other factors most. Therefore, if the internet vendors pay more attention and make the online service strategies well. The improvement in both criteria will lead to the improvement in other criteria as well. Thus, we suggest internet vendors benefit from interactive websites that provide online customer service and enhance the efficiency of data collection by effectively managing the information customers require, therefore ensuring the quality of information customers receive. With a detailed understanding of customers’ needs, employees can provide customers with the products and services they require most. This is the most important task in the e-business and it must be therefore prioritized for improvement. In addition, vendors can communicate product knowledge and proclaim their business philosophies to customers, enabling customers to gain a clear understanding of vendors and subsequently reinforcing trust in vendors. More importantly, they help customers resolve complaints by sufficient job training and actively establish forum platforms for customers to exchange their product reviews and experiences, thereby reducing the likelihood of customers becoming victims of fraud.

We know from Table 7 that criteria weights differ. By combining DEMATEL and ANP method, we found “Transaction security” which is weighted 0.1699 is the main force impacting consumers who are wondering whether to shop on the internet. When consumers purchase online they sometimes also take the “Trust” (0.1587) and “Privacy risk” (0.1414) into consideration. Our results show that online shopping security is the greatest concern for customers; this is in agreement with the results of previous studies. Indeed, certain risks, though seemingly minor, have, in reality, a much greater impact on customers’ purchase attitude. Naturally, online shoppers may hesitate about making purchases through the internet if they doubt about the security when providing and sending their personal or financial information on the public networks.

In any circumstances, the e-retailers must provide an absolute safe and secure means for transactions of personal and financial information through the internet. No deceitful sales or false transactions should be allowed. Therefore, we suggest the e-retailers should enhance online shopping websites, provide clear explanations regarding the sharing of personal information and strictly comply with the provisions to protect personal information. Furthermore, the protection of credit card information is a significant responsibility that should be prioritized for all online shopping website providers; any negative events involving the leak of credit card information will lead to immediate legal disputes. Customers will instantly stop using the website and issue serious complaints. In sum, customers hope to receive a rapid and reliable service when problems are encountered. The improvement of the service quality regarding privacy is a pressing issue, and companies who can make immediate resource investments in this regard will attract customers and increase their satisfaction.

## Conclusion

In an ever-increasingly competitive market, it is important to understand how consumers make a series of decisions regarding information search and data transmission in a Web-base. Although many excellent studies have been devoted to online shopping, none, so far, is able to explain the simultaneous interactions between the various factors, and effectively analyze the real influential weights of all the criteria. The DANP method demonstrated a useful decision making model, which helps to clarify the complicated problems and rank the priority in this study. The three major contributions of this study are summarized as follows: (1) Identify the influential dimensions and criteria through a lot of literature reviews and experts’ opinions, provided by the experienced regular purchases and e-shopping stores operations. This not only produced useful results, but also can act as a reference in this industry. (2) According to the results, DEMATEL analysis can separate complex factors and display them in a causal diagraph, which manifestly provides decision makers with perceivable and comprehensive information to focus easily and thus develop a strategy as reference for the industry. In our study, “Responsiveness” and “Communication and Interaction” are the major issues requiring urgent attention. Since they are causal items, they should be improved directly. From the survey, internet vendor can gain a better understanding of key factors that influence their e-business. (3) This study illustrates the proposed approach combining DEMATEL with ANP to deal with the complexity caused by this interdependence and priority of dimensions and criteria obtained by applying the DANP method in the field of online shopping behavior. Top 3 in the sequence of improvement priorities were as follow: transaction security, trust and privacy risk. Using the ranked lists resulting from the DANP weighting, managers can most effectively compare marketing strategies in order to promote internet sales. In sum, the case study has shown that the DANP method can correctly indicate the effects of internet vendor criteria and identify those that need to be improved with priority. The DANP method can not only detect the dependent relationships and feedback in real complex systems, but also identify a priority sequence among the dimensions and criteria. It may be valuable to both practitioners and researchers as well as for internet vendors that are attempting to expand the management of e-commerce.

Of course, no single method is perfect, and none is reliably able to outperform all other methods in handling all kinds of problems. Nonetheless, the DANP method seems to be useful and effective analyzing complex problem clusters. The DANP method should also provide a paradigm for other industries and stimulate further future research in the area of systematically examining complex decision-making issues with interdependent criteria. The present study inevitably has some limitations calling for further research. First, only small expert samples were surveyed in the case study, and the results drawn from their views may not be fully reflecting the general users’. Second, 12 criteria were considered in this study. It is believed that different industry may be associated with different criteria and the consumers may have different decision-making processes. It is worthwhile to perform cases study for different industry in order to uncover new criteria and to attempt other promising decision-making processes. Third, other new DEMATEL-based hybrid methods (e.g., Tsai and Chou 2009; Hu et al. 2009; Yang and Tzeng 2011) have been found in literature. Developing a hybrid DEMATEL-based framework incorporated with other proper methods can be another avenue for future study.
